# Rosenmüller's Fossa and the Middle Ear

**Published:** 1911-02-11

**Authors:** Macleod Yearsley

**Affiliations:** Senior Surgeon to the Royal Ear Hospital; Medical Inspector to London County Council Deaf Schools, etc.


### Hospital Clinics.

### ROSENMÜLLER'S FOSSA AND THE MIDDLE EAR.

By MACLEOD YEARSLEY, F.R.C.S., Senior Surgeon to the Royal Ear Hospital; Medical Inspector to London County Council Deaf Schools, etc.

**A clinical Lecture delivered at the Royal Ear Hospital on January 4, 1911.**

Rosenmüller's fossa is that lateral recess of the pharynx which lies behind the trumpet-shaped opening of the Eustachian tube, its anterior wall forming the so-called Eustachian cushion. At birth it scarcely exists, the Eustachian prominences being only slightly raised above the surrounding tissues. In the adult, however, it is comparatively deep, rich in glandular tissue, and subject to considerable individual variations in size.

It might be thought that the relation of Rosenmüller's fossa to the middle ear is obvious to every surgeon, considering its close connection with the pharyngeal orifice of that tube, which places the cavity of the tympanum in direct communication with the outer air. Yet, curiously enough, its literature is comparatively meagre, and its importance in otology seems to have been overlooked in quite a remarkable way. If a half-dozen text-books be examined the references to this little anatomical recess will be found to be most scanty.

In spite of this neglect, the fossa of Rosenmüller is one of the most important parts of the upper respiratory tract and its adnexa with which the otologist can have to deal. It is frequently to be found, in both children and adults, to contain pathological tissue. Such tissue is more constant than many would expect, and is a most important factor in the causation and maintenance of morbid conditions of the middle ear tract.

To most students of otology Rosenmüller's fossa merely suggests the ever-present subject of adenoids, and it is true that these growths, or, rather, these hypertrophies, are one of the most important factors in any study of the pathological fossa of Rosenmüller. Therefore I propose to offer you a few remarks upon the adenoid conditions which may be met with in this recess of the naso-pharynx.

The pharyngeal tonsil resembles the faucial tonsils in structure very closely. It extends from the middle line on each side to the fossa of Rosenmüller, which forms a well-marked depression behind the Eustachian orifice, separating the latter from the tonsil. The agglomerate glands are most numerous behind the Eustachian cushions—that is to say, in Rosenmüller's fossæ themselves—and they are also closely grouped together on the upper surface of the soft palate. Now adenoids are not only enlargements of the pharyngeal tonsil, but also of the closed follicles which lie in the mucous membrane of the posterior surface of the vault and the postero-lateral nasopharyngeal walls. If a series of removed adenoids be examined, it will be seen that they can be segregated into at least three groups. The first, which has been called the "soft" adenoid, forms a smooth mass, spreading over the greater part of the naso-pharynx, so soft that it feels almost semi-fluctuating to the examining finger, and which is very considerably influenced by atmospheric conditions and by the physical condition of the child. Microscopic examination will show that that form of "adenoid" is composed almost entirely of lymphoid tissue, very pliable, and covered with a thin epithelial and an ill-defined submucous layer. This is the variety of hypertrophy which, apparently due to an over-development of lymphoid tissue, is easily broken up by the mere pressure of the finger-nail.

The next kind of adenoid to which I would draw your attention is the œdematous variety, in which there is very little increase in the actual gland itself, but the enlargement depends upon venous stasis and œdema. This form is smooth and tense, but easily compressible. It is practically a pharyngeal tonsil engorged and congested, and is directly associated with intestinal irritation and circulatory irregularities. Such a naso-pharyngeal condition is frequently observed in children who are the hosts of some intestinal parasite.

Thirdly, there is the hyperplastic hypertrophy—the "hard" adenoid, in which, beside the increase in the lymphoid constituents, there is connective tissue overgrowth as well. These adenoids often have a well-formed mucous membrane, and are firm and lobulated to the touch.

Now, of these three varieties of hypertrophy of the pharyngeal tonsil, it is the first which is most to be blamed for pathological conditions of the fossa of Rosenmüller. The normal pharyngeal tonsil should atrophy physiologically somewhere, roughly speaking, between the tenth and fifteenth year. When, however, it hypertrophies in the form of a "soft" adenoid, and is not removed surgically, although the large mass in the vault of the post-nasal space may atrophy more or less, the portions extending into the lateral recesses on either side very frequently remain, and may be felt easily by digital examination. Experience has taught me that this condition behind the Eustachian cushions may last into adult life, forming a very active etiological factor in recurrent attacks of Eustachian tube inflammation and lateral pharyngitis. Investigations by American physicians and surgeons (who appear to recognise the importance of Rosenmüller's fossa more than we in the Old World do; at any rate, the majority of the few papers which have been written upon the subject hail from the United States), who have endeavoured to trace the focal source of cervical and bronchial glandular infection to tonsils and adenoids, have pointed to the lateral pharyngeal recess as the culprit in many instances. When one remembers that this fossa is deep, and, in the cases I have indicated, full of friable, succulent, lymphoid tissue, it does not require much reflection to recognise what an ideal cultivation place it makes for micro-organisms. Not only does this initiate and keep up a chronic inflammatory condition, leading to infection of the Eustachian tube and all its results, but the lymphoid tissue mechanically interferes with the action of the muscles which open the tube, and this adds to the obstruction to tympanic ventilation. Nor can there be any doubt that this condition may give rise to difficulties in the venous return from the tympanum and internal ear, resulting in troublesome tinnitus.

Let me offer you two cases which illustrate some of these points. The first is a boy, aged ten, who has had, since the age of four years, recurrent attacks of acute middle-ear suppuration on the left side, and occasionally on the right side also. Two years ago he saw an aural surgeon, who removed his tonsils and adenoids. He breathed better after the operation, but still has the recurrent otorrhœa "whenever he is run down." Examination with the finger revealed the fact that the fossæ of Rosenmüller were entirely occupied by soft lymphoid tissue, which bled easily. This was thoroughly scraped away, and, although it is over six months since this small operation took place, his ears have been dry. Moreover, some glands in the neck, which previously could be felt, are no longer palpable.

The girl of twenty whom you saw this afternoon illustrates another point. She came some weeks ago complaining of a more or less constant tinnitus, chiefly in the right ear, but sometimes in the left also, of a "rushing" character. She did not think it ever left her, and was much worse on lying down and in damp weather. She was not very deaf, but her hearing varied with the tinnitus. She had been treated by various methods, including inflation, which improved the noises temporarily. Now in a patient like this, or, indeed, with any ear patient, there are two points which should never be lost sight of—first, that most causes of ear disease lie in or just behind the nose; second, that tinnitus such as she described is very often due to venous congestion. This patient's naso-pharynx was examined carefully by posterior rhinoscopy, to which she was very tolerant. The space was fairly free, but rather darker in colour than normal, and her Eustachian cushions appeared to be swollen, and the fossæ of Rosenmüller could not be well seen. She was given gas, and it was then found that both fossæ were blocked by soft tissue, easily scraped out. Her tinnitus improved very quickly, and I purposely did not inflate her until two weeks ago, when the catheter appears to have removed the last traces of her trouble.

But, beside this condition of blocking of Rosenmüller's fossæ by soft lymphoid tissue, which has the double result of mechanical obstruction and micro-organic infection, you will often see another result of adenoids, especially in cases in which operation has been imperfect. Unfortunately some surgeons seem to think that the removal of a large central mass, and the restoration, more or less, of nasal respiration thereby, is all that is needed; they neglect, therefore, to clear the lateral recesses as well, a neglect sometimes fatal to the integrity of the ear as an organ of hearing. The result of this imperfect removal is that one or more bands, recognisable by the posterior rhinoscope, pass from the posterior and upper part of the fossa to the cushion of the Eustachian tube. The same appearance is to be seen in cases who have been the subjects of adenoids in childhood and in whom the hypertrophy has atrophied without removal. These bands hamper the proper movements of the Eustachian tube, and sooner or later cause deafness. They are a standing reproach to the people who think and, what is worse, preach that adenoids can be temporised with, or treated by topical applications. Gentlemen, you might as well sing them topical songs!

Take a case in point. A man, aged 38 years, came to me complaining of slight deafness coming on for the past six months. He had had frequent colds the winter before, and was worse with colds, when he also had slight tinnitus, generally rushing in character (probably venous tinnitus again). Health generally good. Has paracusis loci, which means that he experiences difficulty in recognising the locality of sounds. Both membranes were in-drawn, and the mallei mobile. Tests showed middle-ear deafness in an early stage, with Rinne's reaction positive. High tones heard well, low tones impaired. Acoumeter heard 13 feet both sides; voice 13 feet with the right, 9 feet with the left; whisper 13 feet with the right, 1 foot with the left. The catheter improved the whisper to right 15 and left 12 feet.

He had no nasal obstruction, but slight remains of adenoids, easily visible to posterior rhinoscopy, and bands of adhesion passing across both Rosenmüller's fossæ, but more marked on the left side. The clearance of the adenoid remains and of the adhesions, followed by a course of inflation, restored this man's hearing and got rid of his tinnitus, and he has not relapsed.

Thirdly, there is yet another condition of the fossa of Rosenmüller, which gives a quite definite clinical picture. The patient suffers from variable deafness, the fluctuations of which are influenced by dust and changes in the weather. He often complains of a feeling of "something over" the ears, and there is tinnitus and referred abnormal sensations in the throat. Nothing can be seen with the posterior rhinoscope, but the finger feels that the fossa is studded with small, irregular, scattered masses.

You will note, therefore, that there are three conditions recognisable in the fossa of Rosenmüller, only one of which can be definitely seen by posterior rhinoscopy. This is important, because it shows that it is your duty—an unpleasant duty, especially to the patient—to palpate the fossæ with your finger when you have reason to suspect the other two conditions.

The treatment of the first two conditions is obvious—clearing out. The third, though it requires scraping sometimes, yields very well to applications of iodo-glycerin, or to Mandl's pigment. With such topical applications, made with a cotton-wool swab, must be also given daily nasal douching, done with a proper douche or a Higginson's syringe.

so as to wash out the naso-pharynx. To snuff salt and water or use a boat-shaped irrigator is useless in such cases.

Scraping, in the first two conditions, can usually be done sufficiently with the finger-nail. I prefer to do it under gas, for patients resent this form of assault without an anæsthetic. This small operation not only adequately removes the soft tissue, or even the fibrous bands, but it depletes the engorged blood-vessels and relieves stasis more efficiently than any topical applications, and, for this reason, it is sometimes the best course to pursue in the third of the three conditions I have described. Once recovered from, which takes only two or three days, the ventilation of the middle ear is restored, as the tube, now freed, regains its proper movements.

